# IL-22 Negatively Regulates *Helicobacter pylori*-Induced CCL20 Expression in Gastric Epithelial Cells

**DOI:** 10.1371/journal.pone.0097350

**Published:** 2014-05-13

**Authors:** Jia-Perng Chen, Ming-Shiang Wu, Sung-Hsin Kuo, Fang Liao

**Affiliations:** 1 Institute of Microbiology and Immunology, National Yang-Ming University, Taipei, Taiwan; 2 Department of Internal Medicine, National Taiwan University Hospital and National Taiwan University College of Medicine, Taipei, Taiwan; 3 Department of Oncology, National Taiwan University Hospital, Taipei, Taiwan; 4 Cancer Research Center and Graduate Institute of Oncology, National Taiwan University College of Medicine, Taipei, Taiwan; 5 Institute of Biomedical Sciences, Academia Sinica, Taipei, Taiwan; University Medical Center Freiburg, Germany

## Abstract

*Helicobacter pylori* is a Gram-negative bacterium that infects the human gastric mucosa and causes various gastric diseases. *H. pylori* infection induces the production of inflammatory chemokine CCL20 in gastric mucosa and leads to gastric inflammation. Given that the IL-22/IL-22R axis plays a critical role in the regulation of homeostasis and inflammation of epithelial cells at barrier surfaces, we investigated the effect of IL-22 on CCL20 expression induced by *H. pylori*. We demonstrated that *H. pylori* infection of the gastric epithelia-derived AGS cells significantly induced CCL20 expression and the induction was inhibited by IL-22. Functional analysis of the *CCL20* promoter revealed that the *H. pylori*-induced CCL20 expression required the activation of NF-κB, and that IL-22 inhibited the induction by attenuating NF-κB activation. Knockdown of endogenous STAT3 by either short interfering RNAs or a short hairpin RNA significantly reduced the inhibitory effect of IL-22. Furthermore, STAT3 phosphorylation elicited by IL-22 was crucial for the inhibition of *H. pylori*-induced CCL20 expression. Consistent with the *in vitro* data showing that IL-22 negatively regulated *H. pylori*-induced CCL20 expression in gastric epithelial cells, studies on the tissue sections from patients with *H. pylori* infection also revealed an inverse association of IL-22 expression and CCL20 expression *in vivo*. Together, our findings suggest that IL-22 plays a role in the control of overproduction of the inflammatory chemokine and thus may protect the gastric mucosa from inflammation-mediated damage.

## Introduction

*Helicobacter pylori* is a Gram-negative flagellate bacterium that infects the human gastric mucosa [1]. *H. pylori* infection is asymptomatic in most cases, and can persist for a long period of time. *H. pylori* is thought to be a causative agent for the development of gastric inflammatory diseases including gastritis, gastric carcinoma, and mucosa-associated lymphoid tissue lymphoma (MALToma) [2], [3], in which inflammation plays a critical role. The inflammation is associated with the infiltration of leukocytes as well as the production of inflammatory cytokines and chemokines. It has been widely reported that *H. pylori* infection leads to a Th1 immune response in both human and animal studies [4]–[7]. However, recent studies have demonstrated that *H. pylori* infection is also associated with the production of IL-17, indicating that *H. pylori* infection also elicits the Th17 immune response [8]–[10].

Interleukin-22 (IL-22), a member of the IL-10 family of cytokines, is mainly produced by hematopoietic cells involved in both innate and adaptive immunity [11]. IL-22 is produced by subsets of T cells including Th17 and Th22 [12], [13], γδ T cells [14], and subsets of NK cells [15], [16] in humans. Recently, a subset of innate lymphocyte cells (ILCs) was also reported to produce IL-22 [16], [17]. IL-22 targets cells through the IL-22R receptor, which is composed of IL-22R1 and IL-10R2 [18]–[20]. Of note, IL-22R1 is not expressed in hematopoietic cells, but exclusively expressed in the skin, respiratory and digestive tissues [21], suggesting that IL-22 plays a pivotal role in host defense and immune response at epithelial barriers. IL-22 receptor activation is mediated by STAT3 activation [19], [22]. The functionality of IL-22 in the regulation of intestinal and skin immunity has been widely studied [11]. Interestingly, IL-22 enhances the expression of antimicrobial proteins in bacteria infection, suggesting that IL-22 may play a role in host defense against bacterial infection in skin and gut [23], [24]. The involvement of IL-22 in gastric epithelial cells infected with *H. pylori* has not yet been investigated, although it is suggested by several recent observations. Firstly, IL-22 is produced by Th17 cells [12] which have been shown to mediate *H. pylori* infection [8]–[10]. Secondly, IL-22 mRNA expression was reported to be up-regulated in mice infected with *H. felis* [25]. And lastly, we recently identified five single nucleotide polymorphisms (SNPs) of *IL-22* that were significantly associated with *H. pylori*-induced gastric MALToma (unpublished data). Taken together, IL-22 likely plays a role in *H. pylori*-induced gastric diseases.

Chemokine receptor CCR6 is expressed on immature dendritic cells, B cells, memory and effector T cells, Th17 in particular [26]–[30]. Very recently, CCR6 expression has been detected in subsets of innate lymphoid cells [31], [32], important immune cells regulating intestinal immunity. CCR6 plays important roles in gut mucosal immunity [33]–[36]. CCL20, the only chemokine ligand for CCR6, is highly associated with gastrointestinal homeostasis and inflammation [34]–[36]. The CCR6/CCL20 axis is important for the regulation of gut inflammation [34]–[36] and for the formation of isolated lymphoid follicles (ILFs) in the intestine [35], [37]. Several reports have shown that CCL20 was detected in gastric mucosa infected by *H. pylori* [38]–[41]; however, the molecular mechanism on the induction of CCL20 by *H. pylori* in gastric epithelial cells remains to be determined.

In this study, we investigated the mechanism on the induction of CCL20 by *H. pylori* in a gastric epithelial cell line-AGS. In addition, given that CCL20 and IL-22 are highly involved in barrier immunity, we investigated the interplay between CCL20 and IL-22 in gastric epithelial cells infected with *H. pylori*. We found that *H. pylori* infection significantly induced CCL20 expression mediated by NF-κB activation and that the *H. pylori*-induced CCL20 expression was significantly inhibited by IL-22.

## Methods and Materials

### AGS gastric epithelial cell line and *H. pylori* culture

AGS cells (human gastric adenocarcinoma epithelial cell line from ATCC) were grown in the RPMI 1640 medium (Gibco, GrandIsland, NY) supplemented with 10% heat-inactivated FBS (Gibco) and 2 mM L-glutamine (Gibco) and maintained at 37°C in a humidified-atmosphere of 5% CO_2_. *H. pylori* NTUH-C1 strain was isolated from a patient with duodenal ulcer in National Taiwan University Hospital (Taipei, Taiwan). The strain is a naturally competent clinical isolate that was *cagA* ^+^ and *vacA* ^+^ [42]. The bacteria were grown on CDC anaerobe 5% sheep blood agar plate (BD Bioscience) under microaerophilic conditions at 37°C for 16 h, harvested and resuspended in PBS. The bacteria were then diluted with the cell culture medium for further experiments.

### Real time PCR analysis for *CCL20*

Total RNAs were isolated from AGS cells using the Trizol reagent (Life Technologies, Grand Island, NY) and reverse transcribed into cDNA using the SuperScript III First-Strand Synthesis System (Life Technologies) according to the manufacturer's protocol. Real-time PCR for *CCL20* was performed using the TaqMan Gene Expression Assays purchased from Applied Biosystems (Foster City, CA). The PCR cycling protocol entailed 1 cycle at 95°C for 10 min, followed by 40 cycles of 95°C for 15 s and 60°C for 1 min. The human *GAPDH* was used as an endogenous control. The resultant PCR products were measured by Real-time PCR 7500 (Applied Biosystems). The 2^−ΔΔCt^ method was used to quantify the relative changes in *CCL20* expression (ABI PRISM 7700 Sequence Detection System, User Bulletin 2, Applied Biosystems, 1997).

### Construction of *CCL20* luciferase reporter genes

Segments of the *CCL20* promoter from −862 to +71 (short form) and −3491 to +71 (long form) were PCR-amplified from human genomic DNA extracted from MOLT-4 cells using the following primers (forward primer: 5′-GAGAGTTCTTATACTGCCTTA-3′ and 5′- TTCCTAGTTTGTTGAGTGTT-3′ for the short form and the long form, respectively; common reverse primer: 5′-TGGTTTTTAGCTCAAAGAAC-3′) and cloned into the pGL3-Basic firefly luciferase reporter vector (Promega, Mannheim, Germany) to generate pGL3-862 and pGL3-3491 *CCL20* reporter constructs. Reporter constructs containing sequentially truncated fragments (−394, −204, −150, −112, −91 and −50 to +71) of the *CCL20* promoter region were generated in a similar manner as described for generating pGL3-862 and pGL3-3491. Mutant reporter constructs containing targeted substitutions in the C/EBP, NF-κB and SP-1 binding sites were generated using the site-directed mutagenesis kit (Stratagene, La Jolla, CA) with the following primers (C/EBP, 5′-GATGACATGATGGGGCCAGGTTATACCTGGGGAAAACCCCATGTGGCAAC-3′; NF-κB, 5′-GGGCCAGTTGATCAATGGGGAAGGGGCCATGTGGCAACACGC-3′; SP-1, 5′-CCATGTGGCAACACGCATTCTGTGTACATTCCC-3′).

### Cell transfection and luciferase assay

AGS cells were seeded in 12-well plates (3×10^5^/well) and transiently transfected with 0.2 µg of *CCL20* reporter constructs, truncated *CCL20* reporter constructs, mutant *CCL20* reporter constructs, or an NF-κB-luciferase reporter plasmid (pGL4.32[luc2P/NF-κB-BE/Hygro], Promega) using Lipofectamine 2000 (Invitrogen, Carlsbad, CA) according to the manufacturer's instructions. To normalize the transfection efficiency, 10 ng of the pRL-TK Renilla luciferase control vector (Promega, Madison, WI) was cotransfected with each test construct. Five hours after transfection, Lipofectamine 2000 was removed and replaced with complete RPMI 1640 medium. After 16-h culturing, AGS cells were washed three times with PBS and cultured in serum-free RPMI 1640 medium containing 0.5% BSA plus 2 mM L-glutamine for 16 h to reduce the luciferase background levels. AGS transfectants were then pretreated with 10 ng/ml IL-22 (R&D Systems, Minneapolis, MN) for 1 h followed by *H. pylori* infection at a MOI of 15. After the addition of *H. pylori* into the AGS transfectants, the culture plates were subjected to centrifugation at 500 × *g* for 5 min at room temperature to synchronize the infection. After 6-h infection, cells were washed twice with PBS and lysed in 200 µl passive lysis buffer (Promega, Madison, WI). Firefly and Renilla luciferase activities in AGS cell lysates were measured simultaneously using the Dual-Luciferase Reporter Assay System (Promega) according to the manufacturer's instruction and an Optocomp II luminometer (MGM Instruments, Hamden, CT).

### Preparation of cytosolic and nuclear extracts for EMSA analysis

AGS cells were lysed in a lysis buffer (10 mM HEPES pH 7.9, 1.5 mM MgCl_2_, 10 mM KCl, protease inhibitor cocktail, 0.5 mM DTT and 0.6% NP-40) followed by centrifugation at 12,000 × *g* for 5 min at 4°C. The supernatants (cytosolic fractions) were collected and stored at −80°C. The pellets were further solubilized in an extraction buffer (20 mM HEPES pH 7.9, 1.5 mM MgCl_2_, 420 mM NaCl, protease inhibitor cocktail, 25% glycerol), kept on ice for 15 min, and centrifuged at 12,000 × *g* for 5 min at 4°C. The supernatants (nuclear fractions) were collected and stored at −80°C.

### Electrophoretic mobility shift assay (EMSA)

A double-stranded oligonucleotide containing the consensus NF-κB binding site was prepared by the synthesis of complementary oligonucleotides spanning the sequence from −87 to −57 of the *CCL20* promoter (5′- ATCAATGGGGAAAACCCCATGTGGCAACACG-3′). To anneal, equal moles of the complementary oligonucleotides were mixed, heated to 95°C and gradually cooled down to 37°C on a heating block. The double-stranded oligonucleotide was end-labeled with [γ-^32^P] ATP (PerkinElmer) using T4 polynucleotide kinase to a specific activity of 2 to 4×10^7^ cpm/µg. In a 10-µl reaction, the nuclear extract (10 µg) in the binding buffer (20 mM HEPES pH 7.9, 1 mM DTT, 2.5 mM MgCl_2_, 40 mg/ml BSA, 50 mM KCl, 2 µg poly dI:dC) was incubated with the probe (1×10^4^ cpm) at room temperature for 30 min. The probe-protein complexes were separated from the free probe on a 6% native polyacrylamide gel in the TGE buffer (25 mM Tris, 189 mM glycine, 1 mM EDTA) and visualized by autoradiography. Competition assays were performed by incubating nuclear extracts with labeled probes in the presence of 100-fold molar excess of unlabeled oligonucleotides for 30 min at room temperature. To perform supershift experiments, nuclear extracts were incubated with labeled probes in the presence of 0.4 µg of an antibody against NF-κB p65 (sc-372, Santa Cruz Biotechnology) or NF-κB p50 (sc-114, Santa Cruz Biotechnology) for 1 h at room temperature. Preimmune rabbit IgG was used as a control antibody.

### Chromatin Immunoprecipitation assay (ChIP)

The ChIP assay was performed using the Magna Chip A kit (Upstate Biotechnology, Lake Placid, NY) according to the manufacturer's instructions. Briefly, AGS cells were treated with 1% formaldehyde at room temperature for 15 min. Glycine was added to a final concentration of 0.125 M to stop the cross-linking reaction. The cells were collected and lysed followed by centrifugation at 2000× *g* for 5 min. The pellets were collected and resuspended in the nuclear lysis buffer followed by sonication to shear DNA to approximately 500 to 1000 bp fragments using Bioruptor UCD-200 (Diagenode, Liege, Belgium). The cross-linked chromatin was subjected to immunoprecipitation with a rabbit anti-p65 antibody (Cell signal, Danvers, MA) or an isotype antibody overnight at 4°C followed by the addition of protein A magnetic beads (Upstate Biotechnology). After 5-h incubation at 4°C, the immunoprecipitates were then sequentially washed with low salt, high salt, LiCl and finally TE buffer. The washed immunoprecipitates were then subjected to cross-link reversal in the elution buffer at 65°C overnight followed by removal of the beads using a magnetic separator. DNAs in the supernatants were purified and subjected to quantitative PCR analysis using primers designed from the NF-κB consensus binding site in the *CCL20* promoter (forward, 5′–CAGGATTCTCCCCTTCTCAAC-3′; reverse, 5′ –GGGATGGCCCTATTTATAGCA-3′) and SYBR Green fluorescent dye (Bio-Rad). The immunoprecipitated DNAs from each sample were normalized to the input DNA.

### Small interfering RNA (siRNA)

Two siRNAs targeting human *STAT3* were purchased from Qiagen (Gaithersburg, MD). To knockdown endogenous STAT3, AGS cells were plated in 6-well plates (3×10^5^/well) followed by the transfection of siRNA (100 pmole/well) using Lipofectamine 2000 (Invitrogen, Carlsbad, CA). After 5-h incubation, the transfectants were collected and plated in 12-well plates (3×10^5^/well). About 36-h post-transfection, the AGS cells were infected with *H. pylori* in the absence or presence of IL-22 for 6 h. The knockdown efficiency of STAT3 in the cells was determined by Western blotting with an antibody specific for STAT3. The membrane was stripped and re-probed with an antibody to β-actin. The culture supernatants were collected for the detection of CCL20 concentration by ELISA.

### Lentivirus mediated knockdown of STAT3

Two pLKO.1 based constructs TRCN0000020841 (sh-STAT3#7) and TRCN0000020842 (sh-STAT3#8) expressing specific human STAT3 shRNA were obtained from the National RNAi Core Facility, Academia Sinica, Taipei, Taiwan. The recombinant lentiviruses were generated by co-transfection of the pLKO.1 constructs along with pCMVΔR8.91 (packaging plasmid) and pMD.G (envelope plasmid) into 293 T cells. Infectious lentivirus was harvested at 40 and 64 h after transfection. To determine the STAT3 knockdown efficiency, AGS cells were transduced with the recombinant lentivirus for 24 h. The transduced cells were further selected by 5 µg/ml puromycin (Sigma) for 48 h. The knockdown efficiency of STAT3 in cells was determined by Western blotting analysis. For the *H. pylori* infection experiments, the transduced AGS cells were seeded in 12-well plates (3×10^5^/well) and fasted overnight. The fasted AGS cells were pretreated with or without 10 ng/ml IL-22 for 1 h followed by infection with *H. pylori* at a MOI of 15. After infection for 6 h, the concentration of CCL20 in the supernatants was determined by ELISA.

### Overexpression of wild-type STAT3 or dominant negative STAT3 in AGS cells with silenced endogenous STAT3

Plasmids encoding wild type STAT3 (pCMV2-FLAG-STAT3) and dominant negative STAT3 (pCMV2-FLAG-STAT3DN) were generated by cloning the coding regions of wild-type or dominant negative STAT3 (Y705F) into pFLAG-CMV2. AGS cells were transfected with si-RNA#b together with either pCMV2-FLAG-STAT3 or pCMV2-FLAG-STAT3DN. At 36-h post-transfection, the transfectants were infected with *H. pylori* in the presence or absence of IL-22 followed by the determination of CCL20 concentration in cell culture supernatants at 6-h post-infection.

### Measurement of CCL20 production by ELISA

CCL20 in the cell culture supernatants was measured by ELISA (R&D system) according to the manufacturer's protocol.

### Western blot analysis

AGS cells were lysed in the Lysis-M Reagent (Roch, Mannheim, Germany) containing protease inhibitor cocktail (Roch) and phosphatase inhibitor cocktail (Sigma, St. Louis, MO). Fifty micrograms of cell lysate proteins were separated under reducing conditions on a 7.5% SDS-polyacrylamide gel and proteins were then transferred onto nitrocellulose membranes (Perkin Elmer, Waltham, MA). The membranes were blocked with 5% milk in TTBS (150 mM NaCl, 100 mM Tris pH 7.5, 0.1% Tween 20) for 1 h before incubated with anti-phosho-STAT3 (Cell signal Technology, Beverly, MA) at 4°C overnight, followed by three washes. The membrane was then incubated with peroxidase conjugated goat anti-rabbit antibody for 1 h at room temperature followed by three washes, and finally subjected to chemiluminescence using the ECL reagent (Perkin Elmer, Waltham, MA). The same membrane was subsequently stripped and blotted with anti-STAT3 (Santa Cruz Biotechnology) or anti-β-actin (Signa-Aldrich).

### Functional blockade of IL-22 receptor

AGS cells were pretreated with 2 µg/ml of a neutralizing antibody against IL-22R1 (AF2770; R&D Systems, Minneapolis, MN) for 1 h before being infected with *H. pylori* in the absence or presence of IL-22.

### Ethics statement and immunohistochemistry (IHC)

Tissue samples were collected from patients with *H. pylori*-induced MALToma at National Taiwan University Hospital with written informed consent. The study was approved by National Taiwan University Hospital Research Ethics Committee (IRB protocol: NTUH-9561703044). Tissue samples were subjected to IHC analysis for detecting CCL20 and IL-22 expression. Formalin-fixed paraffin embedded sections at a thickness of 4 µm were deparaffinized with xylene followed by rehydration with a graded descending series of alcohol. After antigen retrieval by heating in a 0.1M citrate buffer at pH 6, the endogenous peroxidase activity was blocked by 3% H_2_O_2_. The sections were then incubated in 2.5% normal blocking serum for 30 min followed by the incubation with a primary antibody to CCL20 (AF360; R&D Systems, Minneapolis, MN) or IL-22 (ab18499, Abcam, Cambridge, MA) for overnight. Protein expression was detected using the Super Sensitive Polymer-HRP IHC Detection System (BioGenex Laboratories Inc., San Ramon, CA). The immunostaining procedure was performed according to Maae et al [43]. The percentages of CCL20 or IL-22 positive cells were averaged to yield an immunohistologic score ranging from 0% to 100%. The results were classified into two groups (positive-immunostaining group and negative-immunostaining group) according to the intensity and extent of staining. For the negative-immunostaining group, either no staining was present (staining intensity score = 0) or positive staining was detected in less than 10% of the cells (staining intensity score = 1); for the positive-staining group, moderate or strong immunostaining was present in 10% to 30% (staining intensity score = 2) or more than 30% of the cells (staining intensity score = 3).

### Statistical analysis

The unpaired Student *t* test was used to evaluate the significance of the difference between two experimental results. For the analysis of patients' tissue sections for IHC staining, the Fisher's exact test was used for statistical analysis due to small sample size (n = 24). A *p* value <0.05 was considered statistically significant.

## Results

### IL-22 inhibits the expression of CCL20 induced by *H. pylori* infection in gastric epithelial cells

**Figure 1:**
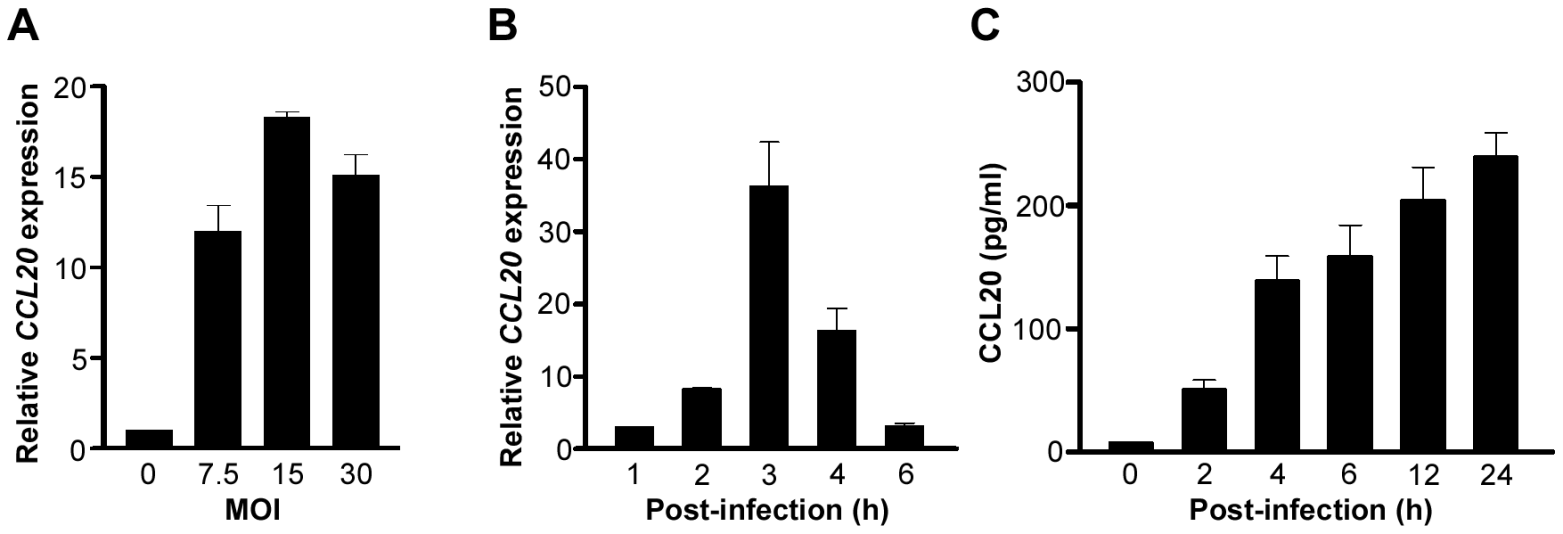
*H. pylori* infection induces CCL20 expression in AGS cells. ***A***, *CCL20* mRNA expression in AGS cells infected with *H. pylori* at indicated MOI was analyzed by real-time PCR at 3-h post-infection. ***B***, *CCL20* mRNA expression in AGS cells infected with *H. pylori* (MOI = 15) was analyzed by real-time PCR at indicated times. ***C***, The cell culture supernatants from AGS cells infected with *H. pylori* were collected at the indicated times and the CCL20 protein level was determined by ELISA. Data represent the mean ± SEM and reflect one representative of three independent experiments.

Given that CCL20 plays a role in gut mucosal inflammation [34]–[36] and that CCL20 expression is detected in mucosal tissues infected by *H. pylori* [38], [40], [41], we examined whether CCL20 expression was induced in the human gastric epithelial cell line-AGS cells upon *H. pylori* infection. Infection of AGS cells with *H. pylori* at various MOI resulted in *CCL20* induction. The optimal MOI for *CCL20* induction was 15 (Fig. 1A), and *CCL20* mRNA expression peaked at 3-h post-infection (Fig. 1B). Consistent with the induction of *CCL20* mRNA, CCL20 protein secretion was also increased in AGS cells infected with *H. pylori* (Fig. 1C). These results demonstrate that *H. pylori* infection induces CCL20 expression at both mRNA and protein levels in gastric epithelial cells.

**Figure 2:**
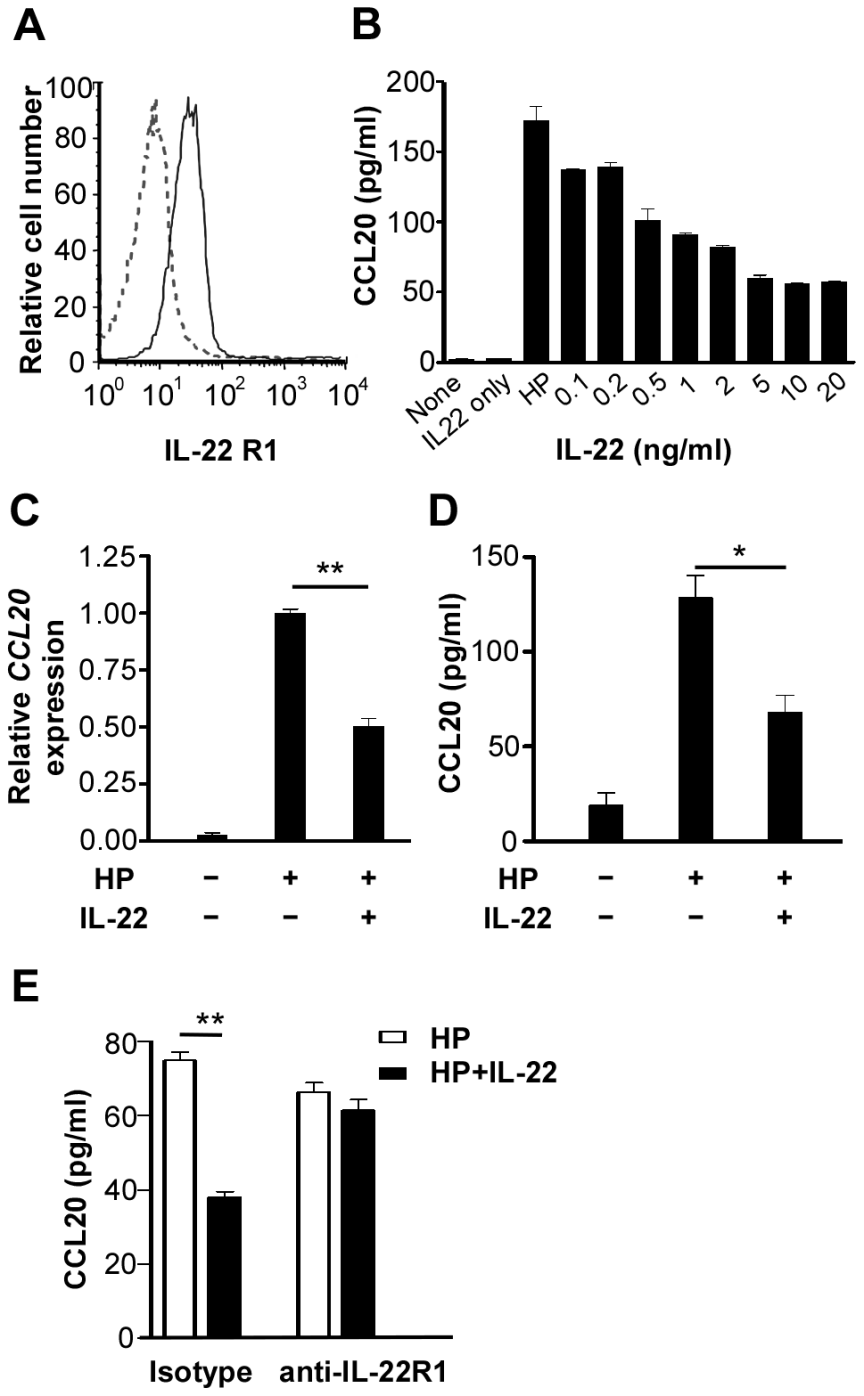
Stimulation of AGS cells with IL-22 leads to the inhibition of *H. pylori-*induced CCL20 expression. ***A***, AGS cells were stained with an isotype control antibody (dashed line) or an anti-IL-22R1 antibody (solid line) conjugated with allophycocyanin and analyzed by flow cytometry. ***B*** *,* AGS cells were infected with *H. pylori* (HP) in the presence of various concentration of IL-22 for 24 h and the CCL20 concentration in the culture supernatants was determined by ELISA. ***C-D***, AGS cells were infected with *H. pylori* in the presence or absence of IL-22 and the *CCL20* mRNA in the cells (***C***) and CCL20 protein in the culture supernatants (***D***) were determined by real-time PCR and ELISA, respectively. ***E***. AGS cells were pretreated with a neutralizing antibody against IL-22R1 (2 µg/ml) for 1 h followed by the *H. pylori* infection in the absence or presence of IL-22 for 6 h. The CCL20 concentration in the culture supernatants was determined by ELISA. Data represent the mean ± SEM from three independent experiments. **, *p*<0.005; *, *p*<0.02 for *H. pylori* + IL-22 *versus H. pylori* only.

IL-22 is known to play a role in mucosa immunity [11], [44]–[46] and targets cells through the IL-22 receptor, which is composed of two receptor chains, IL-22R1 and IL-10R2 [19], [20], [22]. IL-22R1 is a receptor specific for IL-22 and exclusively expressed in barrier tissues, while IL-10R2 is broadly expressed in a variety of tissues [22]. Given that both IL-22 and CCL20 are highly involved in the regulation of mucosal immunity and/or inflammation, we investigated the interplay between IL-22 and CCL20 in *H. pylori*-infected AGS cells. We first demonstrated by FACS analysis that AGS cells expressed IL-22R1 (Fig. 2A). The presence of IL-22 during *H. pylori* infection of AGS cells led to the inhibition of *H. pylori*-induced CCL20 expression in a dose-dependent manner (Fig. 2B). IL-22 (10 ng/ml) significantly inhibited *H. pylori*-induced *CCL20* expression at both mRNA (*p*<0.005) (Fig. 2C) and protein (*p*<0.02) levels (Fig. 2D) and the inhibitory effect was about 40% for both. To demonstrate that the inhibition of *H. pylori*-induced CCL20 was indeed mediated by IL-22, we functionally blocked IL-22R in AGS cells with a neutralizing anti-IL-22R1 antibody before infecting cells with *H. pylori* in the absence or presence of IL-22 and CCL20 secretion was measured in culture supernatants. Blocking the IL-22 receptor completely abolished the inhibitory effect of IL-22 on *H. pylori*-induced CCL20 expression (Fig. 2E). Similarly, IL-22R1 knockdown by siRNA also significantly reduced the inhibitory effect of *H. pylori*-induced CCL20 expression by IL-22 (Figure S1).

### The inhibition of *H. pylori*-induced CCL20 expression by IL-22 is mediated by transcriptional regulation

**Figure 3:**
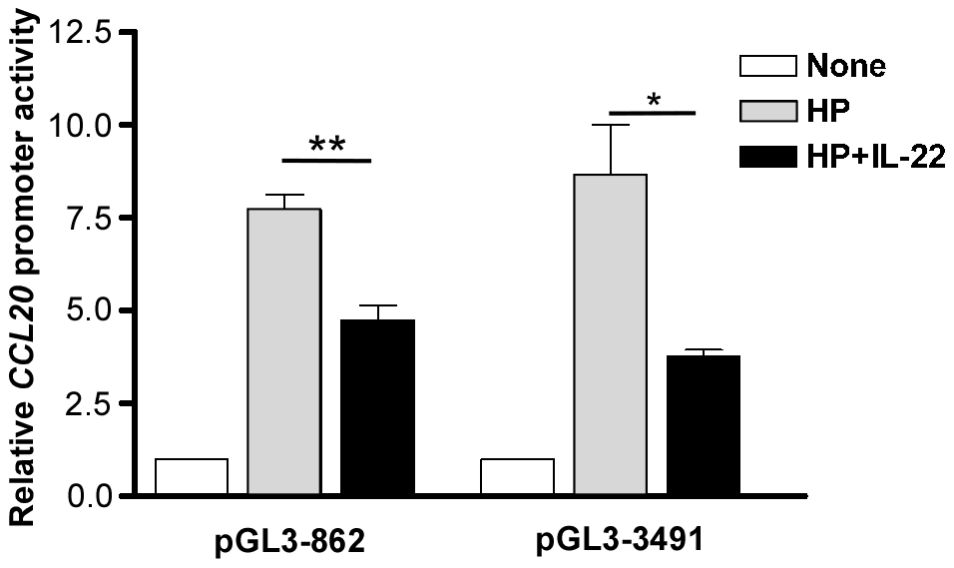
IL-22 inhibits *CCL20* promoter reporter activities in AGS cells infected with *H. pylori*. AGS cells were co-transfected with the *CCL20* luciferase reporter construct pGL3-862 or pGL3-3491 along with pRL-TK *Renilla* luciferase plasmid. At 48 h after transfection, cells were infected with *H. pylori* in the presence or absence of IL-22. Luciferase activities were normalized to the expression of the cotransfected pRL-TK *Renilla* luciferase plasmid. The activity from each construct is presented as the relative luciferase activity of *H. pylori*-infected cells versus uninfected cells (open bar; set as 1). Data represent the mean ± SEM from three independent experiments. *, *p*<0.05; **, *p*<0.01 for *H. pylori* + IL-22 *versus H. pylori* only.

We next examined whether the IL-22-attenuated *H. pylori*-induced CCL20 expression was mediated by reducing the transcriptional activity of *CCL20*. To identify the region of the *CCL20* promoter responsible for *CCL20* gene transactivation in response to *H. pylori*, we generated two pGL3-CCL20 reporter constructs, pGL3-862 and pGL3-3491 containing a 933-bp (from −862 to +71) and a 3562-bp (from −3491 to +71) segment, respectively, of the human *CCL20* 5′-flanking region linked upstream to the firefly luciferase coding sequence. The pGL3-CCL20 constructs were transfected into AGS cells followed by *H. pylori* infection in the presence or absence of IL-22 (10 ng/ml). Cells transfected with pGL3-862 expressed similar relatively luciferase activities as compared with cells transfected with pGL3-3491 (Fig. 3). Furthermore, IL-22 exerted similar inhibitory effects on luciferase activities in cells transfected with pGL3-862 and pGL3-3491 (*p*<0.01 for pGL3-386; *p*<0.05 for pGL3-3491) (Fig. 3). These results suggest that the *CCL20* promoter region responsible for IL-22-attenuated *CCL20* induction is located within 862 bp upstream of the transcription initiation site.

### *H. pylori*-induced CCL20 expression via NF-κB activation is inhibited by IL-22

**Figure 4:**
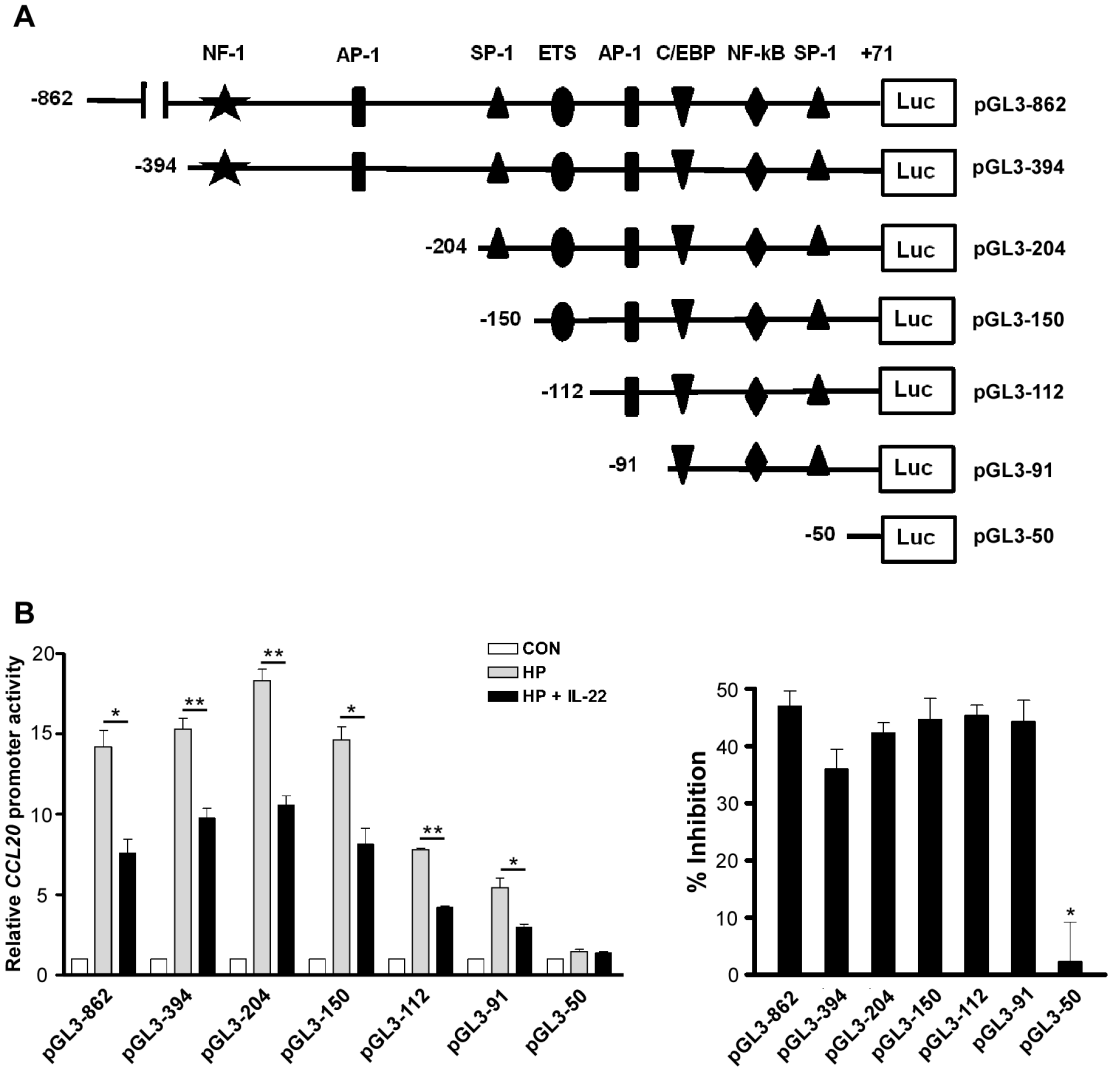
The *CCL20* promoter region (nucleotides -91 to -50) is critical for the IL-22-attenuated *CCL20* expression. ***A***, Schematic representation of luciferase reporter constructs containing different lengths of the *CCL20* promoter. The locations of putative binding sites for various transcription factors are indicated at top. ***B***, Inhibition of *H. pylori*-induced *CCL20* promoter activity by IL-22. Left panel, AGS cells were co-transfected with the indicated *CCL20* promoter constructs and pRL-TK *Renilla* luciferase plasmid. At 48 h after transfection, cells were infected with *H. pylori* in the presence or absence of IL-22. Luciferase activity was normalized to the expression of a co-transfected pRL-TK *Renilla* luciferase plasmid. The activity of each construct is presented as relative luciferase activity of *H. pylori*-infected cells versus uninfected cells (open bar; set as 1). *, *p*<0.02; **, *p*<0.005 for *H. pylori* + IL-22 *versus H. pylori* only. Right panel, the inhibitory efficiency (% inhibition) was calculated as follows: [(*CCL20* promoter activity in the absence of IL-22 − *CCL20* promoter activity in the presence of IL-22) / *CCL20* promoter activity in the absence of IL-22]×100. Data represent mean ± SEM from three independent experiments. *, *p*<0.05 for pGL3-50 *versus* other reporter constructs.

**Figure 5:**
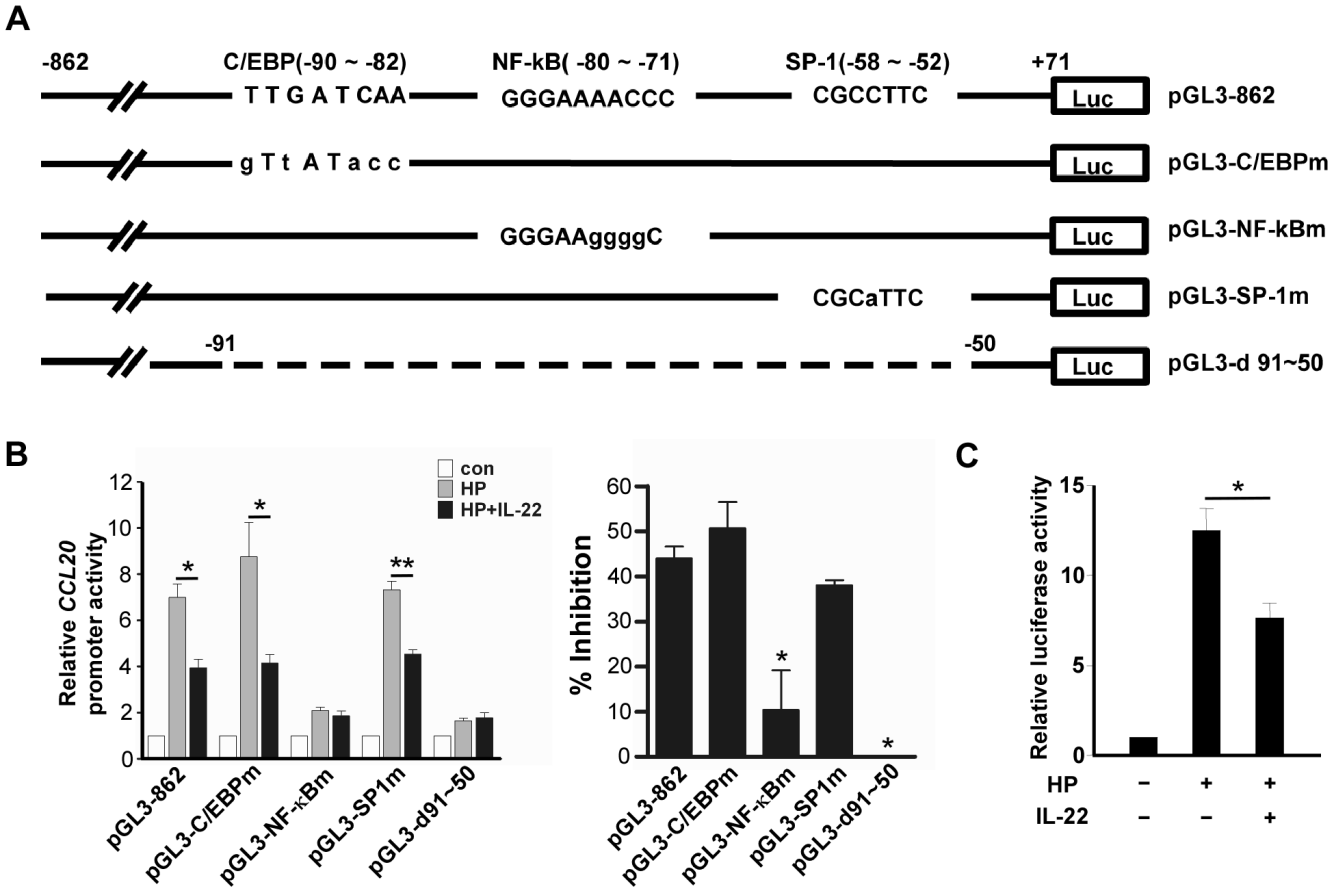
The NF-κB binding site in the *CCL20* promoter is critical for the IL-22-attentuated *CCL20* expression. ***A***, Schematic representation of mutated or deleted *CCL20* reporter constructs. Nucleotide substitutions are indicated in lower case and the deleted region is indicated as dash line. ***B***, Inhibitory effects of IL-22 on *CCL20* promoter activities. Left panel, AGS cells were co-transfected with the indicated *CCL20* promoter constructs and pRL-TK *Renilla* luciferase plasmid. At 48 h after transfection, cells were infected with *H. pylori* in the presence or absence of IL-22 and the relative luciferase activity was measured. Data represent the mean ± SEM from three independent experiments. *, *p*<0.05, **, *p*<0.005 for *H. pylori* + IL-22 *versus H. pylori* only. Right panel, the inhibitory efficiency (% inhibition) were calculated as described in Fig. 4B. *, *p*<0.05 *versus* pGL3-862, pGL3/EBPm or pGL3-SPm. ***C***, Inhibitory effects of IL-22 on a promoter containing NF-κB binding sites. AGS cells were co-transfected with pGL4.32 NF-κB luciferase reporter and pRL-TK *Renilla* luciferase plasmid. At 48 h after transfection, cells were infected with *H. pylori* in the presence or absence of IL-22 and the relative luciferase activity was measured. Data represent the mean ± SEM from three independent experiments. *, *p*<0.05 for *H. pylori* + IL-22 *verses H. pylori* only.

The *CCL20* promoter region has been reported to contain several putative binding sites for transcription factors, including NF-1, Ets, AP-1, C/EBP, NF-κB and SP1 [47]–[49]. These binding sites are schematically shown in Fig. 4A. To identify the *cis*-acting elements that are responsible for IL-22-attenuated *CCL20* induction, we generated a series of constructs with sequential deletions from the 5′ end of the *CCL20* promoter in pGL3-862 to positions -394 (pGL3-394), -204 (pGL3-204), -150 (pGL3-150), -112 (pGL3-112), -91 (pGL3-91), and -50 (pGL3-50) (Fig. 4A) and transfected these constructs into AGS cells followed by *H. pylori* infection in the presence or absence of IL-22. IL-22 treatment reduced *H. pylori*-induced CCL20 reporter activities about 40% in AGS cells transfected with all *CCL20* deletion constructs except pGL3-50 (Fig. 4B), suggesting that the element (s) responsible for the IL-22-attenuated *CCL20* induction was located within the -91 to -50 region which contained putative binding sites for three transcription factors, namely C/EBP, NF-κB and SP-1. To determine which transcription factor(s) was responsible for the IL-22-attenuated *CCL20* induction, we performed site-directed mutagenesis on pGL3-862 to generate constructs pGL3-C/EBPm, pGL3-NF-κBm, and pGL3-SP-1m which contained nucleotide substitutions in the consensus binding site for C/EBP, NF-κB, and SP1, respectively (Fig. 5A). We also deleted nt -91 to -50 in pGL3-862 to generate a deletion construct pGL3-d91-50. These constructs were transfected into AGS cells followed by *H. pylori* infection in the absence or presence of IL-22, and the luciferase activities in the transfected cells were measured. IL-22 was able to inhibit luciferase activities in *H. pylori*-infected AGS cells transfected with either pGL3-C/EBPm or pGL3-SP-1m, but not in AGS cells transfected with pGL3-NF-κBm or pGL-d91-50 (Fig. 5B). These results suggest that NF-κB was involved in the IL-22-attenuated *CCL20* induction. To further confirm this notion, an NF-κB luciferase reporter construct containing five copies of an NF-κB response element was transfected into AGS cells followed by *H. pylori* infection in the presence or absence of IL-22. As shown in Fig. 5C, *H* *. pylori*-induced NF-κB luciferase activity was inhibited by the presence of IL-22 (*p*<0.05). Taken together, results from the reporter assays clearly indicate that the NF-κB consensus binding site in the *CCL20* promoter is critical for conferring the inhibitory effect of IL-22 on *H. pylori*-induced *CCL20* expression.

**Figure 6:**
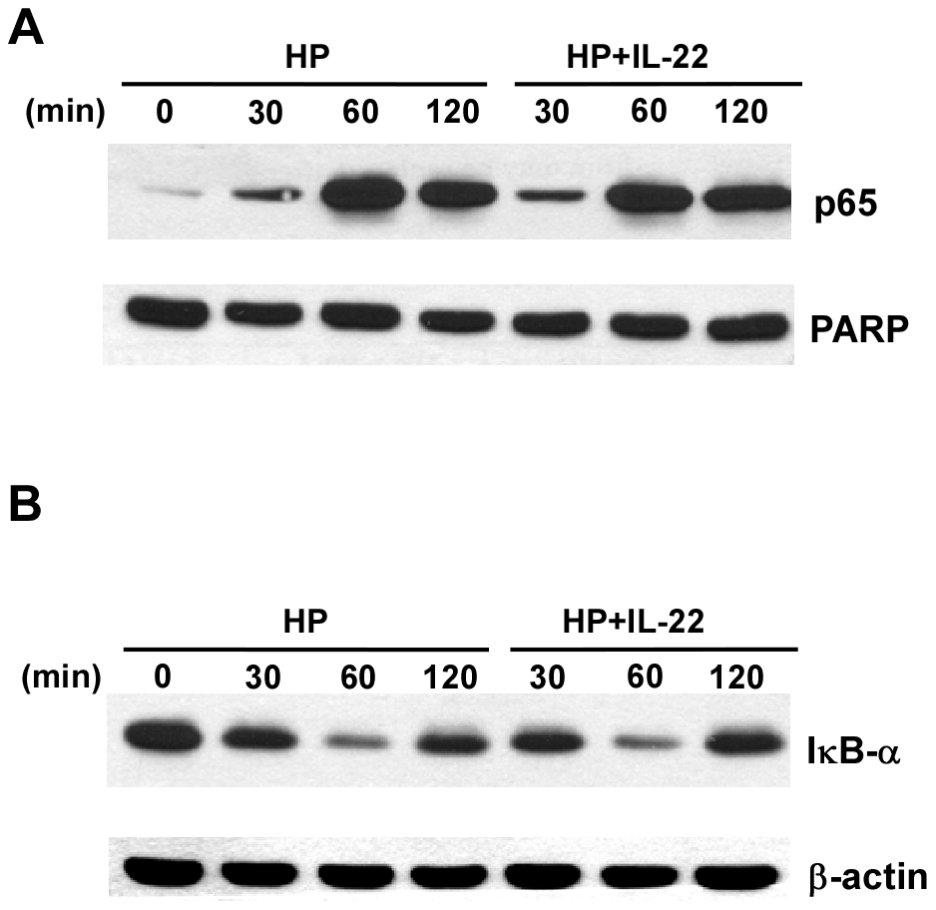
IL-22 does not affect the nuclear translocation of NF-κB or degradation of IκB. AGS cells were infected with *H. pylori* in the presence or absence of IL-22. The cells were lysed at indicated times and the lysates were subjected to nuclear fractionation. ***A***. The nuclear fractions were run on a SDS-PAGE gel followed by immunoblotting with anti-NF-κB p65. The membrane was re-probed with an antibody against PARP, a nuclear marker. ***B***. The cell lysates were subjected to SDS-PAGE followed by immunoblotting with an antibody to IκB-α. The membrane was stripped and re-probed with anti-β-actin.

We then examined whether IL-22 prevented the degradation of IκB or the nuclear translocation of NF-κB, leading to reduced levels of NF-κB. Western blotting showed that *H. pylori* infected AGS cells with or without IL-22 treatment had similar levels of NF-κB in the nuclei (Fig. 6A) and that IL-22 did not affect the degradation of IκB (Fig. 6B). The results suggested that the inhibitory effect of IL-22 on *H. pylori*-induced *CCL20* expression was not due to impaired NF-κB activation, but more likely due to reduced binding of NF-κB to the NF-κB consensus binding site in the *CCL20* promoter after nuclear translocation of NF-κB.

**Figure 7:**
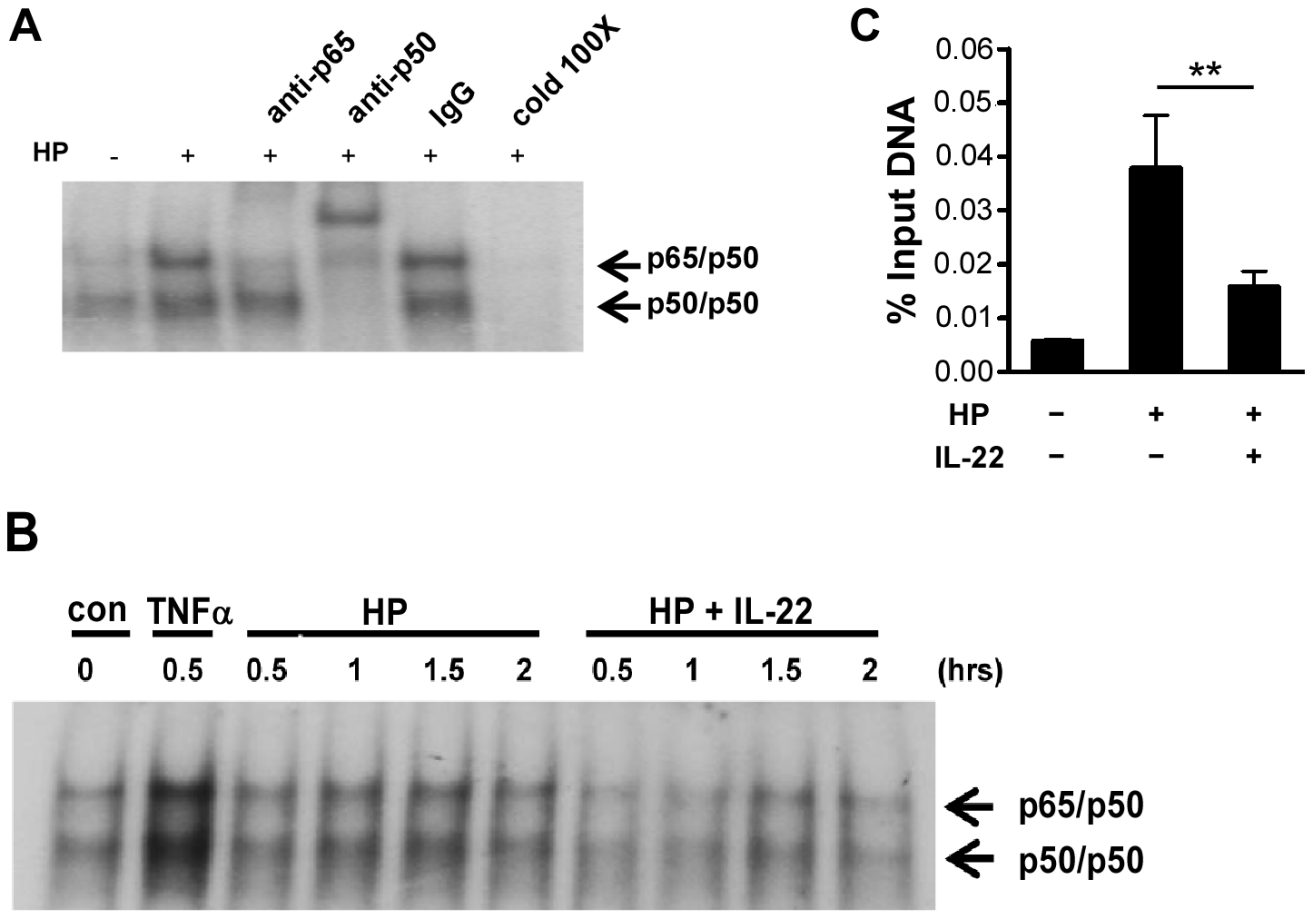
IL-22 reduces the binding of NF-κB to the *CCL20* promoter in *H. pylori*-infected AGS cells. ***A***, EMSA supershift study of CCL20-specific NF-κB activation in *H. pylori*-infected AGS cells. Nuclear extracts isolated from AGS cells without (−) or with (+) *H. pylori* infection were incubated with a ^32^P-labelled probe containing the NF-κB binding sequence in the *CCL20* promoter and either excess cold probe (cold 100X), control antibody (IgG), or an antibody to NF-κB p50 or p65 subunit and then subjected to EMSA analysis. ***B***, Effects of IL-22 on the binding of NF-κB to the *CCL20* promoter. AGS cells were infected with *H. pylori* in the absence or presence of IL-22 and nuclear extracts were isolated at the indicated times post-infection and subjected to EMSA analysis. TNF-α treated AGS cells were used as a positive control for NF-κB activation. ***C***, ChIP assay for the binding of NF-κB p65 to the endogenous *CCL20* promoter. Chromatin was prepared from AGS cells infected with *H. pylori* in the absence or presence of IL-22. NF-κB p65 binding to the *CCL20* promoter was determined by ChIP assay using an anti-p65 antibody for immunoprecipitation. Immunoprecipitated DNA from each sample was assayed by PCR for the presence of the *CCL20* promoter and the result was normalized to the input DNA control. Data represent the mean ± SEM from four independent experiments. **, *p*<0.02 for *H. pylori* + IL-22 *versus H. pylori* only.

We further conducted EMSA to demonstrate that NF-κB binding to the NF-κB consensus binding site in *CCL20* promoter was critical for IL-22-attenuated *CCL20* induction. Nuclear extracts from AGS cells infected with *H. pylori* in the absence or presence of IL-22 were incubated with a ^32^P-labelled oligonucleotide probe comprising the NF-κB binding site in the *CCL20* promoter, and the presence of NF-κB in the binding complexes was detected by supershift reactions with antibodies specific to p50 or p65 NF-κB subunits. As shown in Fig. 7A, the radioactive DNA-protein complexes contained both p50/p50 homodimer and p50/p65 heterodimer, and the presence of excess cold probe diminished the complexes formation, demonstrating the specificity of the binding. The binding of both NF-κB p50/p50 and NF-κB p50/p65 to the NF-κB consensus binding site was reduced when nuclear extracts from AGS cells infected with *H. pylori* in the presence of IL-22 were used (Fig. 7B). Consistent with the EMSA results, the ChIP assay showed IL-22 significantly reduced the interaction of NF-κB p65 with the *CCL20* promoter in AGS cells (*p*<0.02) (Fig. 7C).

### The STAT3 signaling plays a role in the inhibitory effect of IL-22 on *H. pylori*-induced CCL20 expression

**Figure 8:**
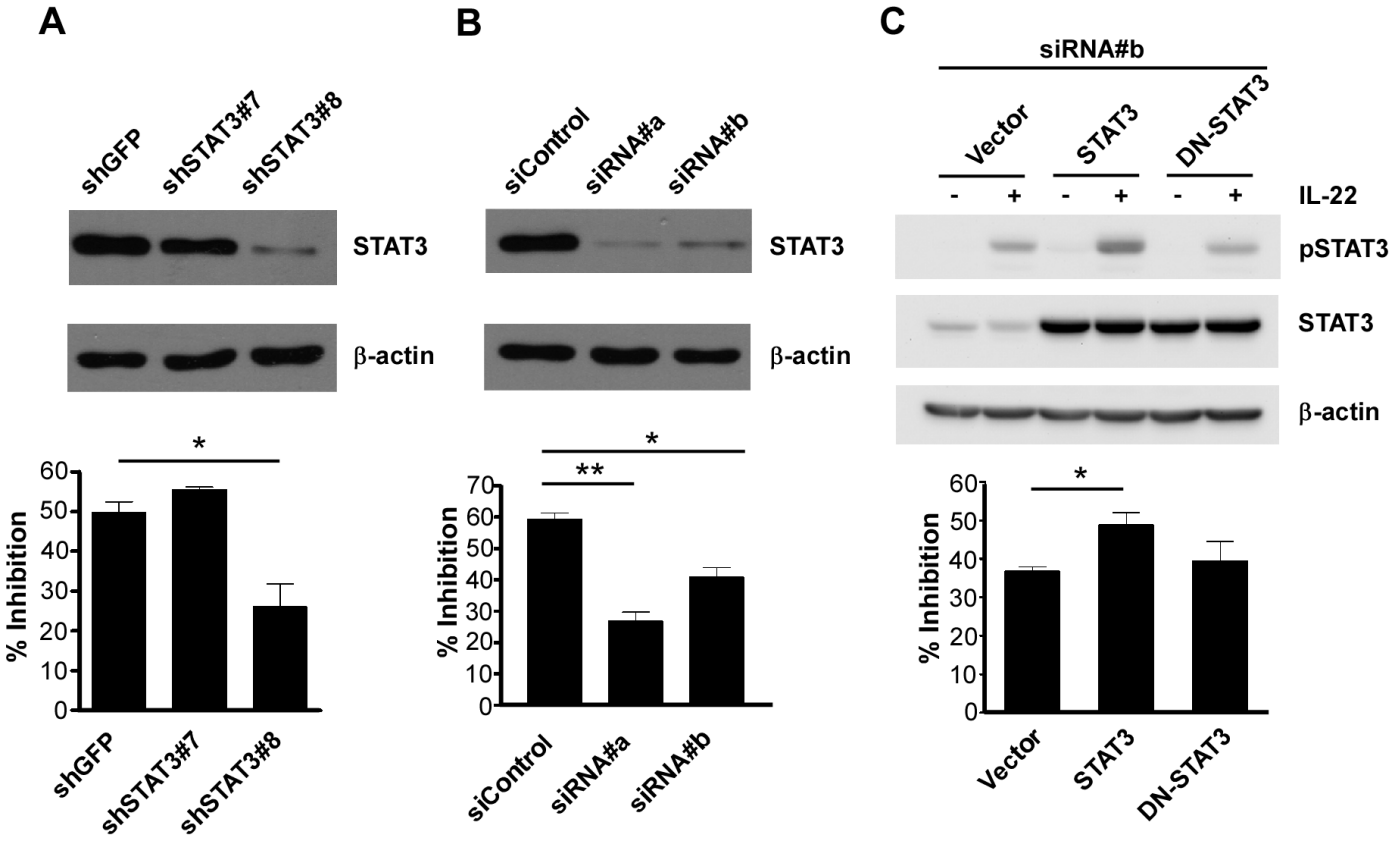
Knockdown of STAT3 reduces the inhibitory effect of IL-22 on *H. pylori*-induced CCL20. ***A***, Knockdown of STAT3 with shRNAs. AGS cells were infected with lentivirus carrying STAT3-specific shRNA clone 7 or clone 8 for 24 h. After puromycin selection for another 48 h, total cell lysates were subjected to immunoblotting with anti-STAT3. The membrane was stripped and re-probed with anti-β-actin (upper panel). The cells with STAT3 knockdown were infected with *H. pylori* in the presence or absence of IL-22, and the culture supernatants were collected for the determination of CCL20 concentration by ELISA. The lower panel shows the inhibitory efficiency (% inhibition), which was calculated as follows: [(CCL20 concentration in *H. pylori*-infected cells without IL-22 − CCL20 concentration in *H. pylori*-infected cells with IL-22) / CCL20 concentration in *H. pylori*-infected cells without IL-22]×100. Data represent the mean ± SEM and reflect one representative of four independent experiments. *, *p*<0.02 *versus* sh-GFP. ***B***, Knockdown of STAT3 with siRNAs. Similar to (***A***) except that siRNA#a and siRNA#b were used to knockdown STAT3. The lower panel shows the inhibitory efficiency, which was calculated as described in (***A***). Data represent the mean ± SEM and reflect one representative of two independent experiments. *, *p*<0.002 *versus* si-control; **, *p*<0.0001 *versus* si-control. ***C***, AGS cells were transfected with siRNA#b together with either the expression vector encoding wild-type STAT3 (WT-STAT3) or the expression vector encoding dominant-negative STAT3 (DN-STAT3). Total cell lysates were subjected to immunoblotting with anti-phospho-STAT3 and the membrane was stripped and re-probed with anti-STAT3 (upper panel). At 36-h post-transfection, the transfectants were infected with *H. pylori* in the presence or absence of IL-22 followed by the determination of CCL20 in cell culture supernatants 6-h post-infection (lower panel). The inhibitory efficiency was calculated as described in (***A***). Data represent the mean ± SEM from three independent experiments. *, *p*<0.01 *versus* vector.

IL-22 targets cells by binding to the IL-22R heterodimer to exert its biological function via STAT3 activation [22]. To investigate whether the IL-22-attenuated CCL20 induction was mediated by STAT3, we silenced the endogenous STAT3 expression by transfecting STAT3 shRNA or siRNA into AGS cells followed by *H. pylori* infection in the absence or presence of IL-22. Western blotting showed that STAT3 was silenced efficiently by shSTAT3#8 but not shSTAT3#7 (Fig. 8A, upper panel), and as expected, the AGS cells transfected with shSTAT3#8 showed significantly reduced IL-22-attenuated CCL20 induction (shSTAT3#8 vs. sh-GFP, *p*<0.02) (Fig. 8A, lower panel). Similarly, both siRNA#a and siRNA#b significantly silenced endogenous STAT3 expression in AGS cells (Fig. 8B, upper panel) and reduced the inhibitory effect of IL-22 on the *H. pylori*-induced CCL20 expression (siRNA#a vs. si-control, *p*<0.0001; siRNA#b vs. si-control, *p*<0.002) (Fig. 8B, lower panel). Taken together, these results indicate that knockdown of STAT3 significantly reduced, but not completely blocked, the IL-22-attenuated CCL20 induction, suggesting that the inhibitory effect of IL-22 on the *H. pylori*-induced CCL20 expression in AGS cells is dependent, in part, on STAT3 signaling.

Phosphorylation of STAT3 is critical for the IL-22 receptor signaling [22]. We thus examined whether STAT3 phosphorylation was required for IL-22-attenuated CCL20 induction. We first silenced the endogenous STAT3 using siRNA#b which targeted 3′UTR of STAT3 and then transfected cells with a plasmid encoding either the wild-type STAT3 (WT-STAT3) or a dominant-negative phosphorylation-deficient STAT3 (DN-STAT3), and these transfectants were then infected with *H. pylori* in the absence or presence of IL-22 followed by the determination of *H. pylori*-induced CCL20 expression. Although AGS cells transfected with WT-STAT3 showed an increased level of phosphorylated STAT3 (pSTAT3) and significant inhibitory effect of IL-22 on the *H. pylori*-induced CCL20 expression compared to cells transfected with the cloning vector (WT-STAT3 vs. vector, *p*<0.01), cells transfected with DN-STAT3 showed no difference in both the level of pSTAT3 and inhibitory effect on CCL20 expression (Fig. 8C). These results suggest that phosphorylation of STAT3 is required for the IL-22-attenuated CCL20 induction.

### IL-6 shows less inhibitory effects than IL-22 on the *H. pylori*-induced CCL20 expression in AGS cells due to weaker and transient phosphorylation of STAT3

**Figure 9:**
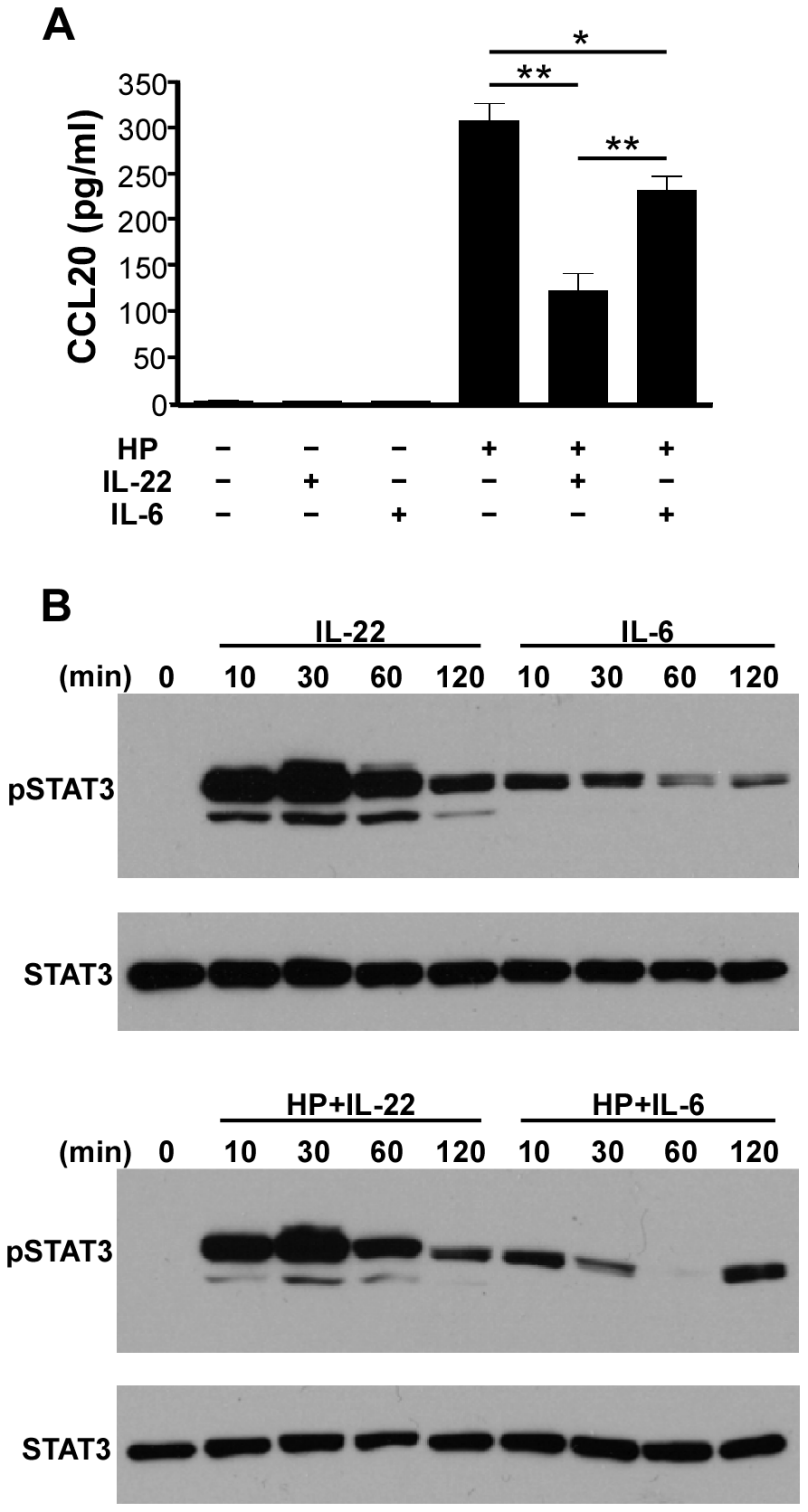
IL-6 elicits lower STAT3 phosphorylation and exerts less inhibition on CCL20 expression than IL-22. ***A***, Inhibitory effects of IL-22 and IL-6 on *H. pylori*-induced CCL20 expression. AGS cells were infected without or with *H. pylori* in the presence or absence of IL-22 or IL-6. The cell culture supernatants were collected 6 h post-infection and subjected to ELISA determination of CCL20 concentration. Data represent the mean ± SEM and reflect one representative of three independent experiments. *, *p*<0.001; **, *p*<0.0001. ***B***, Effects of IL-22 and IL-6 on STAT3 phosphorylation. AGS cells were infected without (upper panel) or with (lower panel) *H. pylori* followed by stimulation with IL-22 (10 ng/ml) or IL-6 (100 ng/ml). At the indicated times, the cells were lysed and subjected to SDS-PAGE followed by immunoblotting with a phospho-STAT3 (tyrosine 705) specific antibody. The membrane was stripped and re-probed with anti-STAT3.

We have shown that IL-22-attenuated CCL20 induction is mediated by STAT3 activation (Fig. 8). IL-6 is known as a pro-inflammatory cytokine and its signaling through STAT3 is found in many inflammatory diseases [50], [51]. We next examined whether IL-6 also inhibited *H. pylori*-induced CCL20 expression. AGS cells were infected with *H. pylori* in the absence or presence of IL-22 or IL-6, and the expression of CCL20 was determined. IL-6 or IL-22 alone did not induce CCL20 expression in AGS cells without *H. pylori* infection (Fig. 9A). Although both IL-6 and IL-22 were capable of inhibiting *H. pylori*-induced CCL20 expression in AGS cells (*H. pylori* +IL-22 vs. *H. pylori*, *p*<0.0001; *H. pylori* +IL-6 vs. *H. pylori*, *p*<0.001), IL-6 elicited significantly less inhibition compared to IL-22 (*H. pylori* +IL-22 vs. *H. pylori* +IL-6, *p*<0.0001) (Fig. 9A). Given that the inhibition of *H. pylori*-induced CCL20 expression in AGS cells involved STAT3, as shown above, we reasoned that the difference in the inhibitory efficiency of IL-6 and IL-22 on the *H. pylori*-induced CCL20 expression might be due to a difference in the kinetics of STAT3 phosphorylation between IL-6 and IL-22 treated cells. We therefore performed Western blotting using an antibody specific for phosphorylated STAT3 (Tyr705) and found that cells treated with IL-22 with or without *H. pylori* infection showed significantly strong and prolonged phosphorylation of STAT3, while cells treated with IL-6 showed rather weak and transient STAT3 phosphorylation (Fig. 9B). Taken together, these results suggest that strong and prolonged STAT3 phosphorylation is likely critical for the inhibition of *H. pylori*-induced CCL20 by IL-22. We constantly observed that in AGS cells infected with *H. pylori* in the presence of IL-6, STAT3 phosphorylation reappeared at 120 min after it declined to almost undetectable levels at 60 min after infection (Fig. 9B). The molecular mechanism attributing to the phenomenon is currently unknown. One possibility is that *H. pylori* CagA might activate STAT3 phosphorylation in the later stage of infection [52] and cause the rebounded STAT3 phosphorylation.

### IL-22 expression is inversely associated with the CCL20 expression in patients with *H. pylori*-induced gastric MALToma

To evaluate whether our findings have any clinical implication, we examined the levels of IL-22 and CCL20 expression in gastric tissue sections from patients with *H. pylori*-induced MALToma by IHC staining (Fig. S2). There was an inverse association between the expression level of IL-22 and that of CCL20 (*p*<0.05, Table 1), suggesting that IL-22 may have a regulatory role *in vivo* in controlling the *H. pylori*-induced CCL20 expression.

**Table 1:** The IL-22 expression is inversely associated with CCL20 expression in gastric mucosa tissues evaluated by IHC.

	CCL20 Negative (N = 13)	CCL20 Positive (N = 11)
IL-22 Negative (N = 14)	5 (35.7%)	9 (64.3%)
IL-22 Positive (N = 10)	8 (80%)	2 (20%)

Gastric mucosa specimens (n = 24) obtained from patients with *H. pylori*-induced MALToma were subjected to immunohistochemistry analysis for detecting CCL20 and IL-22 expression. Samples were assigned positive or negative for the expression as described in the MATERIALS AND METHODS section. Statistical significance of the differences was determined by the Fisher's exact test with *p* = 0.047.

## Discussion

The CCR6/CCL20 axis plays crucial roles in the homeostasis of gut-associated lymphoid tissues and in the regulation of mucosal immunity [35], [36], [53]. Increased CCL20 expression has been observed in gastrointestinal inflammation [35], [36] as well as skin inflammation [54], [55]. Studies have shown that *H. pylori* infection induces CCL20 expression in gastric tissues of human patients or neonatally thymectomized mice, and that CCL20 is selectively produced by gastric epithelial cells [38]–[41]. In this study, we have investigated the molecular mechanism underlying *H. pylori*-induced CCL20 expression by both promoter truncation and mutation assays, and found that a NF-κB consensus binding site in the *CCL20* promoter was crucial for the induction of CCL20 by *H. pylori* infection. This notion was further confirmed by chromatin immunoprecipitation assay showing direct binding of NF-κB to the *CCL20* promoter. Although we demonstrated that the induction of CCL20 expression by *H. pylori* infection is mediated by transcription regulation through NF-κB activation, the upstream molecules utilized by *H. pylori* to trigger NF-κB activation and subsequent induction of CCL20 expression remain elusive. TLR2, TLR4 and TLR5 have been suggested to be responsible for the recognition of *H. pylori* [56] and subsequent mediation of NF-κB activation; however, this notion has been questioned due to the low intrinsic activities of *H. pylori* lipopolysaccharide and flagellin on TLR activation. Additionally, NOD1 has also been reported to sense *H. pylori* and lead to NF-κB activation [57]; however this is also debatable because peptidoglycan does not seem to be the bacterial factor that activates NF-κB [58]. Identifying host factors responsible for NF-κB activation that subsequently leads to CCL20 induction warrants further investigation.

Interestingly, we found that IL-22 was able to regulate *H. pylori*-induced CCL20 expression. Accumulating evidence has shown that IL-22 plays a role in intestinal immunity, particularly in the regulation of intestinal epithelial cells' integrity and restitution [59]. Evidence for this pivotal role of IL-22 in host defense at epithelial barriers is further strengthened by the notion that IL-22 receptor (IL-22R1) is expressed exclusively in barrier tissues, such as skin, respiratory and digestive tissues [21]. IL-22 knockout mice showed impairments in the synthesis of antimicrobial proteins in keratinocytes and epithelial cells of intestine and lung to mediate innate host defense against bacteria [21], [24], [60]. Furthermore, IL-22 knockout mice failed to maintain the integrity and/or restitution of intestinal epithelial cells [59]. On the other hand, despite its protective role, IL-22 has also been reported to be associated with inflammatory diseases in skin [61], [62] and colon [63]. IL-22 has thus been considered to function as a double-edged sword in intestinal inflammatory responses, i.e. pro-inflammatory versus anti-inflammatory responses. The opposite biological consequences of IL-22 observed in intestinal tissues are likely a result of different context of the cells, tissues, cytokine milieu and disease models being used. In the present study, we demonstrated that IL-22R is also expressed in gastric epithelial cells and found that IL-22 inhibits *H. pylori*-induced CCL20, suggesting that IL-22 may serve as a negative regulator for *H. pylori*-induced CCL20, preventing overproduction of inflammatory chemokine-CCL20 from gastric epithelial cells to maintain a balance of immune responses. Additionally, IL-22 has been shown to enhance intestinal epithelial restitution, wound healing and mucus barrier in intestine. Similarly, IL-22 may play a role in epithelial cells recovery repair as well as controlling bacteria infection in gastric tissues. It is plausible that induction of CCL20 in epithelial cells during early phase of *H. pylori* infection may be important for recruiting CCR6-bearing DC, ILC3, and Th17 cells to control the pathogen. However, it is critical to have a “guardian”, in this case IL-22, to prevent overproduction of CCL20 during the late phase of infection leading to tissue damages caused by amplified inflammatory responses.

We further demonstrated that the inhibitory effect of IL-22 on *H. pylori*-induced CCL20 production in gastric epithelial cells is, in part, STAT3-dependent since knockdown of STAT3 in the cells significantly attenuated, but not completely inhibited CCL20 expression. Despite that STAT3 activation by IL-6 has been considered detrimental in intestinal inflammation [64], our study showed that STAT3 activation by IL-22 seems beneficial, preventing overproduction of inflammatory chemokine-CCL20. Several studies have also found that STAT3 activation is able to attenuate inflammation. Welte et al. demonstrated that deficiency of STAT3 in DC or B cells results in enhanced NF-κB activation upon stimulation of TLRs, indicating that STAT3 activation down-regulates NF-κB activation in hematopoietic cells [65]. Yu et al. showed that STAT3 inhibits iNOS transcription by interacting with NF-κB, which serves to control the level of iNOS so as to avoid cytotoxic effects of NO to the host [66]. Our current study demonstrates that STAT3 phosphorylation elicited by IL-22 is able to attenuate CCL20 expression induced by *H. pylori* infection and that *H. pylori* induces CCL20 expression via NF-κB activation, suggesting that STAT3 activation likely interferes with NF-κB activation and subsequently leads to the decrease of CCL20 expression. Given that IL-22-stimulated STAT3 activation in intestinal epithelial cells is critical for mucosal wound healing [59] and that our present study shows that IL-22 attenuated *H. pylori*-induced CCL20 expression in gastric epithelial cells is mediated by STAT3 activation, it is plausible that the IL-22-STAT3 axis plays a protective role in gastric mucosal inflammation.

Although IL-6 signals through STAT3 phosphorylation, we found that IL-6 inhibited *H. pylori*-induced CCL20 less efficiently as compared to IL-22. The discernable difference of STAT3 activation between IL-22 and IL-6 may lie in the duration of STAT3 phosphorylation (Fig. 9). IL-22 induces a strong and sustained STAT3 phosphorylation, while IL-6 induces a weak and transient STAT3 phosphorylation. It seems that sustained STAT3 phosphorylation may be critical for the anti-inflammatory property. It is noted that IL-10, an anti-inflammatory cytokine exclusively targeting hematopoietic cells, also induces strong and sustained STAT3 phosphorylation in hematopoietic cells [67]. If sustained STAT3 phosphorylation is important for the anti-inflammatory effect for IL-22 or IL-10, IL-22 and IL-10 should be able to prolong the half-life of STAT3 phosphorylation more than IL-6. The key molecule that determines the half-life of STAT3 phosphorylation is suppressor of cytokine signaling-3 (SOCS3), which inactivates and degrades STAT3 [67]. The gp130 of IL-6R has docking sites for SOCS3. Neither IL-10R nor IL-22R binds SOCS3 due to the lack of SOCS3 docking site. Therefore, upon IL-10 or IL-22 stimulation in cells, STAT3 is phosphorylated and SOCS3 fails to terminate STAT3 phosphorylation, resulting in prolonged STAT3 phosphorylation. The absence of negative regulatory effects on IL-22 by SOCS3 may be, in part, a critical step in establishing the anti-inflammatory response. The next important question would be how STAT3 mediates IL-22 to exert its anti-inflammatory response in gastric epithelial cells upon *H. pylori* infection. Several *in vitro* studies have demonstrated that STAT3 is able to downregulate NF-κB activity and inhibit the transcription of NF-κB driven genes [65], [66]. Furthermore, STAT3 has been shown to directly interact with NF-κB p65 and serve as a dominant-negative inhibitor of NF-κB activity [66]. We have been unable to demonstrate the interaction of NF-κB and STAT3 in *H. pylori*-infected AGS cells (data not shown). By examining the *CCL20* promoter sequence, we found that there is a putative STAT3 binding sequence, TTN_4–6_AA [68], [69] (from -83 to -90), right before the NF-κB binding site. It is plausible that phosphorylated STAT3 may compete spatially with NF-κB for the binding site and thus interfere with the binding of NF-κB to its consensus binding sequence. Interestingly, it has been reported that unphosphorylated STAT3 facilitates NF-κB activation and subsequently promotes the expression of a chemokine-CCL5 [70]. Our present study shows that IL-22 stimulates a pronged and sustained STAT3 phosphorylation, which may decrease the availability of unphosphorylated STAT3 in cells and reduce NF-κB activation and subsequently CCL20 expression. It is speculated that the sustained phosphorylated STAT3 may act as a transrepressor, which expels NF-κB from the *CCL20* promoter, thus inhibiting the transcription of *CCL20*. The mechanism on how STAT3 inhibits NF-κB activation during *H. pylori* infection warrants further investigation.

Finally, we examined whether the inhibitory effect of IL-22 on *H. pylori*-induced CCL20 expression in gastric epithelial cells can be recapitulated in clinical settings. Gastric specimens of patients with *H. pylori*-induced gastric MALToma showing detectable levels of IL-22 expression tend to have undetectable levels of CCL20 expression, suggesting an inverse association of IL-22 expression and CCL20 expression *in vivo*. Based on the *in vitro* data and the clinical observation in this study, IL-22 appears to play a beneficial and/or protective role in *H. pylori*-induced gastric diseases, preventing overproduction of inflammatory cytokines and maintaining the homeostasis of gastric immunity.

## Supporting Information

Figure S1 **Knockdown of IL-22R1 in AGS cells reduces the inhibitory effect of IL-22 on** ***H. pylori*** **-induced CCL20 expression.** ***A***, AGS cells with IL-22R1 knocked down by siRNA were stained with an isotype control antibody (red) or an anti-IL-22R1 antibody (green) conjugated with allophycocyanin followed by FACS analysis. AGS cells treated with a nonspecific siRNA were used as a control (blue). ***B***, The AGS cells with IL-22R1 knockdown were infected with *H. pylori* in the presence or absence of IL-22, and CCL20 in culture supernatants was determined by ELISA. The % inhibition was calculated as described in Fig. 4B.
(TIF)

Figure S2 **IL-22 expression is inversely associated with the CCL20 expression in patients with** ***H. pylori*** **-induced MALToma.** Gastric mucosa samples from patients with *H. pylori*-induced MALToma were subjected to the detection of CCL20 and IL-22 expression by IHC using the Super Sensitive Polymer-HRP IHC Detection System. Representative immunostaining images of CCL20 (***A***) and IL-22 (***B***) are shown, with panel (a) and panel (b) showing examples of negative and positive staining, respectively.
(TIF)
