# Supplementary figures and images for: IL-22 Negatively Regulates Helicobacter pylori-Induced CCL20 Expression in Gastric Epithelial Cells
Source: PLoS One. 2014 May 13;9(5):e97350. doi: 10.1371/journal.pone.0097350 (PMC4019584; doi:10.1371/journal.pone.0097350)

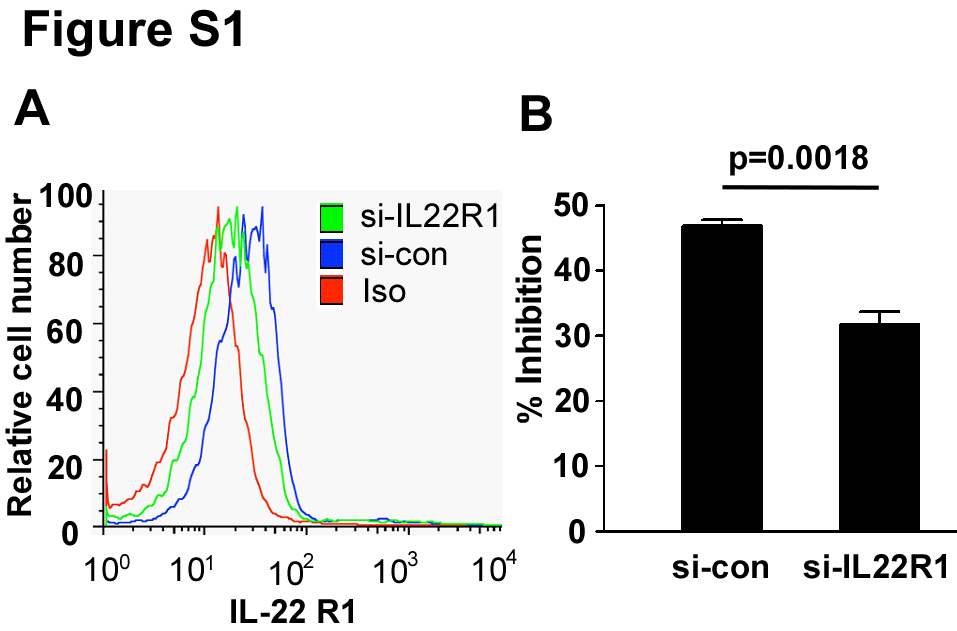

Supplement: Figure S1 — Knockdown of IL-22R1 in AGS cells reduces the inhibitory effect of IL-22 on H. pylori -induced CCL20 expression. A, AGS cells with IL-22R1 knocked down by siRNA were stained with an isotype control antibody (red) or an anti-IL-22R1 antibody (green) conjugated with allophycocyanin followed by FACS analysis. AGS cells treated with a nonspecific siRNA were used as a control (blue). B, The AGS cells with IL-22R1 knockdown were infected with H. pylori in the presence or absence of IL-22, and CCL20 in culture supernatants was determined by ELISA. The % inhibition was calculated as described in Fig. 4B. (TIF) [file pone.0097350.s001.tif]

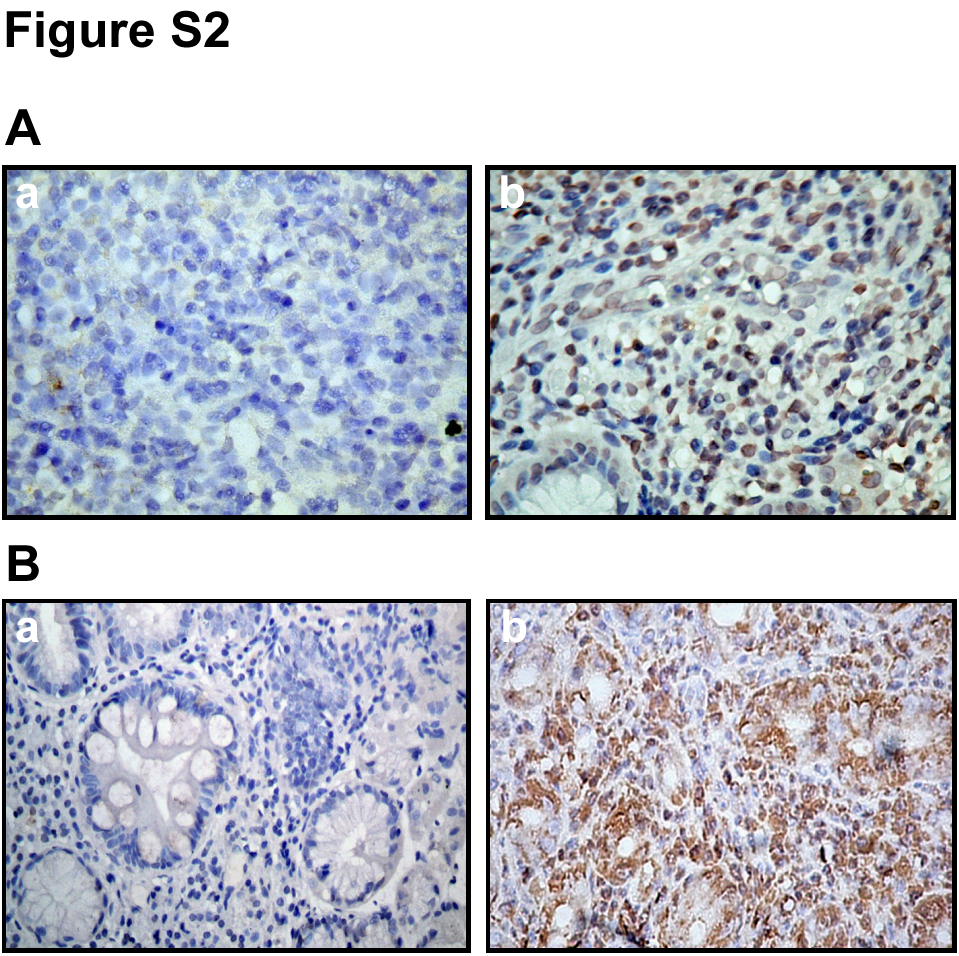

Supplement: Figure S2 — IL-22 expression is inversely associated with the CCL20 expression in patients with H. pylori -induced MALToma. Gastric mucosa samples from patients with H. pylori-induced MALToma were subjected to the detection of CCL20 and IL-22 expression by IHC using the Super Sensitive Polymer-HRP IHC Detection System. Representative immunostaining images of CCL20 (A) and IL-22 (B) are shown, with panel (a) and panel (b) showing examples of negative and positive staining, respectively. (TIF) [file pone.0097350.s002.tif]
